# Editorial: Recent Trends in Morphological Computation

**DOI:** 10.3389/frobt.2021.708206

**Published:** 2021-05-31

**Authors:** Keyan Ghazi-Zahedi, John Rieffel, Syn Schmitt, Helmut Hauser

**Affiliations:** ^1^ Max Planck Institute for Mathematics in the Sciences, Leipzig, Germany; ^2^ Union College, Schenectady, NY, United States; ^3^ Institute for Modelling and Simulation of Biomechanical Systems, University of Stuttgart, Stuttgart, Germany; ^4^ Department of Engineering Mathematics, University of Bristol, Bristol, United Kingdom

**Keywords:** morphological computation, soft robotics, embodied intelligence, robotics, control

**Editorial on the Research Topic**

**Recent Trends in Morphological Computation**

Morphological Computation is a concept that suggests that morphological properties, such as the shape and form of a body, as well as dynamical properties like compliance, resonance, and friction, play a crucial role in the emergence of intelligent behavior in nature. Many biological systems appear to have found clever ways to exploit morphological features of their bodies to improve interaction with their environment. As a result, important functions like sensing, control or even computation are partly outsourced to the body’s morphology. This, in turn, enables biological systems to be extremely robust, energy efficient, and highly adaptive. Clearly, these are all properties that are very desirable for robotics systems as well, especially, if they should operate in complex and noisy environments.

While the role of morphological features in biological systems is well accepted, so far, the translation and application of this concept to robotics remains underexplored. One of the main reasons is that the emphasis on the body as a resource for functionality is in stark contrast to the currently dominating design and control paradigms in robotics, where the body is seen as something that needs to be dominated. The robot’s morphology is seen as part of the problem rather than part of the solution. Current robotic systems use rigid body parts and high torque servo motors to suppress any undesirable morphological behaviors like nonlinearity, underactuation or noise (Hauser et al., 2014). The motivation is that rigid bodies can be captured by very simple models and, therefore, can be easily controlled. However, at the same time this method of robot design might overlook the potential for embedding beneficial functionalities within the body and it overrides any natural movements by using a large amount of energy.

Remarkably, these same complex morphological properties that are avoided in conventional robotic designs often play a key role in the behavior of natural systems, many of which outperform state-of-the-art robots in many real-world tasks. Indeed, the only place where modern robotics systems are better than their biological counterparts is in high precision and fast movements in highly controlled environments like factory floors or research labs. Outside of these conditions, e.g., in our working and living spaces, current robotics design mostly fail. Hence, there is a huge potential for novel robotics systems that, besides the use of digital computational power in form of Artificial Intelligence, use their morphological features to make them more intelligent through Morphological Computation.

Recently, the concept has gained an increased interest due to several technological leaps and the emergent of novel research fields. On one hand additive manufacturing has accelerated and multiplied the possibilities of materials that can be used with 3D printing technology. This is partly also driven by the recent emergence of the field of Soft Robotics, which also takes inspiration from nature and suggests building robots by using a larger variety of materials including soft ones like silicone, rubber, polymers, hydrogels, and many others (Rus and Tolley, 2015). Interestingly, the bodies of soft robots naturally exhibit complex nonlinear dynamics that can be effectively leveraged in the context of Morphological Computation (Rieffel et al., 2009; Rieffel and Mouret, 2018). Another contributing factor to the interest in Morphological Computation is that the field of Biomechanics is now able to provide much more detailed models of skeletal muscle systems, which allow robotics researchers and biologists alike to better understand the contribution of the body to observed behaviors like running, walking or pointing (Haeufle et al., 2014; Wochner et al., 2020; Walter et al., 2021). Furthermore, substantial progress has also been made with understanding and formalizing Morphological Computation in terms of dynamical systems (Hauser et al., 2011; Hauser et al., 2012) and information theory (Haeufle et al., 2014; Zahedi and Ay, 2013; Ghazi-Zahedi et al., 2016). In the context of this research topic, recent trends in Morphological Computation have been detailed in legged locomotion, to understand contributions of passive, soft tissues for a successful and robust motion generation. It has been shown that the known hierarchical organisation of biological control can be analyzed using Morphological Computation as quantification criterium and it has been found that this can result in a reduction of neuronal load. Additionally, a minimalistic scenario, based on the concept of relevant information, how and when embodiment for effective robot performance affects the decision density has been identified.

The benefits of Morphological Computation for robots are significant. However, for it to achieve its full potential, we need to extend the notion of how we build machines. We need to embrace complex nonlinear dynamics and explore how the body, the “brain” and the environment can work together to build the next generation of robots.
